# Diversity of *Escherichia coli* strains involved in vertebral osteomyelitis and arthritis in broilers in Brazil

**DOI:** 10.1186/s12917-016-0762-0

**Published:** 2016-07-14

**Authors:** Juliana Fortes Vilarinho Braga, Nathalie Katy Chanteloup, Angélina Trotereau, Sylvie Baucheron, Rodrigo Guabiraba, Roselene Ecco, Catherine Schouler

**Affiliations:** 1 Departamento de Clínica e Cirurgia Veterinárias, Escola de Veterinárias, Universidade Federal de Minas Gerais, Av. Antônio Carlos, 6627, Campus Pampulha, 30161-970 Minas Gerais, Brazil; 2 ISP, INRA, Université François Rabelais de Tours, 37380 Nouzilly, France

**Keywords:** Broilers, Bacterial infections, APEC, Virulence genes, Pathology, Multidrug-resistant *E. coli*

## Abstract

**Background:**

Locomotor disorders and infections by *Escherichia coli* represent major concerns to the poultry industry worldwide. Avian pathogenic *E. coli* (APEC) is associated with extraintestinal infections leading to respiratory or systemic disease known as colibacillosis. The most common lesions seen in cases of colibacillosis are perihepatitis, airsacculitis, pericarditis, peritonitis/salpingitis and arthritis. These diseases are responsible for significant economic losses in the poultry industry worldwide. *E. coli* has been recently isolated from vertebral osteomyelitis cases in Brazil and there are no data on molecular and phenotypic characteristics of *E. coli* strains isolated from lesions in the locomotor system of broilers. This raised the question whether specific *E. coli* strains could be responsible for bone lesions in broilers. The aim of this study was to assess these characteristics of *E. coli* strains isolated from broilers presenting vertebral osteomyelitis and arthritis in Brazil.

**Results:**

Fifteen *E. coli* strains from bone lesions were submitted to APEC diagnosis and setting of ECOR phylogenic group, O serogroup, flagella type, virulence genes content, genetic patterns by Pulsed Field Gel Electrophoresis (PFGE) and Multilocus Sequence Typing (MLST). In addition, bacterial isolates were further characterized through a lethality test, serum resistance test and antibiotic resistance profile. *E. coli* strains harbored different genetic pattern as assessed by PFGE, regardless of flock origin and lesion site. The strains belonged to seven sequence types (STs) previously described (ST117, ST101, ST131, ST 371 and ST3107) or newly described in this study (ST5766 and ST5856). ECOR group D (66.7 %) was the most frequently detected. The strains belonged to diverse serogroups (O88, O25, O12, and O45), some of worldwide importance. The antibiotic resistance profile confirmed strains’ diversity and revealed a high proportion of multidrug-resistant strains (73 %), mainly to quinolones and beta-lactams, including third generation cephalosporin. The percentage of resistance to tetracycline was moderate (33 %) but always associated with multidrug resistance.

**Conclusions:**

Our results demonstrated that vertebral osteomyelitis and arthritis in broilers can be associated with highly diverse *E. coli* based on molecular and phenotypic characteristics. There was no specific virulence patterns of the *E. coli* strains associated with vertebral osteomyelitis or arthritis. Also, *E. coli* strains were frequently multidrug resistant and belonged to STs commonly shared by APEC and human ExPEC strains.

**Electronic supplementary material:**

The online version of this article (doi:10.1186/s12917-016-0762-0) contains supplementary material, which is available to authorized users.

## Background

*Escherichia coli* is a genetically diverse bacteria comprising non-pathogenic intestinal strains and pathogenic strains responsible for intestinal and extra-intestinal disease [1]. The strains able to cause disease in chickens are known as Avian pathogenic *E. coli* (APEC). APEC is associated with extraintestinal infections leading to respiratory or systemic disease known as colibacillosis. These diseases are responsible for significant economic losses in the poultry industry worldwide, which may occur by decreased hatching rates, mortality, lowered production, carcass condemnation at processing and treatment costs [2]. The most common lesions associated with colibacillosis are perihepatitis, airsacculitis and pericarditis, although other syndromes such as osteomyelitis, arthritis, yolk peritonitis, peritonitis/salpingitis (SPS syndrome), coligranuloma, omphalitis and cellulitis can also be found [3].

Another challenge to modern poultry industry is locomotor disorders, which represent a major economic and welfare problem. Although these disorders may be classified according to underlying pathology as infectious, developmental and degenerative, this classification is difficult since these categories are not mutually exclusive [4]. Infectious conditions such as osteomyelitis, arthritis (or osteoarthritis) and synovitis can be associated with different etiologic agents [3]. Among bacteria, *Staphylococcus* sp. (mainly, *S. aureus*) was isolated from these diseases, although an increase in the incidence of musculoskeletal infection associated with *E. coli* has been reported [5].

Brazil, which is currently the largest exporter and the second largest producer of poultry meat in the world, faces challenges with colibacillosis and locomotor disorders the same form as other countries with relevant poultry industry. There are no data on molecular and phenotypic characteristics of *E. coli* strains isolated from lesions in the locomotor system of broilers, although *E. coli* has been recently isolated from vertebral osteomyelitis cases in Brazil [6]. This raises the question whether specific *E. coli* strain could be responsible for bone lesions in broilers. The aim of our work is to provide data on the phenotypic and molecular characteristics of *E. coli* strains isolated from vertebral osteomyelitis and arthritis cases in broilers from Brazil.

## Methods

### Samples

Fifteen *E. coli* strains isolated between 2012 and 2014 from broilers presenting vertebral osteomyelitis or arthritis at commercial poultry farms in the state of Minas Gerais, Southeast of Brazil, were studied. The broilers were from eight different flocks, which represent seven different farms. They had variable ages and gender. All experimental procedures were approved by the Universidade Federal de Minas Gerais (UFMG), Committee for Ethics in Animal Experimentation (CETEA) under protocol 205/2011.

### Clinical signs and pathology

For clinical examination, broilers presenting locomotor disorders were placed in station and encouraged to move for change in gait and posture assessment. Then, broilers were euthanized by cervical dislocation for necropsy and gross evaluation. The locomotor system was analyzed for size, shape, color, flexibility and breaking strength. The vertebral column of all broilers was sectioned along the longitudinal midline for vertebral body and spinal cord analysis. The free thoracic vertebra was considered as T4. Articulations were analyzed for size and aspects of the synovial fluid in the articular space. Samples for bacterial isolation were collected aseptically from broilers presenting osteomyelitis or arthritis. Tissue sections were fixed in 10 % neutral buffered formalin for 48 to 56 hours. Then, formalin-fixed-vertebral column, intertarsal and femorotibial articulations with lesions were decalcified in 24 % formic acid. For slide preparation, tissues were dehydrated in increasing ethanol concentrations, diaphoanised in xylene, embedded in paraffin to obtain 4-μm thick serial sections and then stained with hematoxylin-eosin (HE) and Goodpasture for further analysis under a light microscope.

### Bacterial isolation and identification

Swabs of the lesions were inoculated onto two blood agar (BA) plates and one MacConkey agar (MCK) plate. One BA plate was incubated in microaerophilic conditions at 37 °C for 24 to 72 h, while the others were incubated at the same temperature and time under aerobic conditions. After the initial growth, morphology of isolated colonies was characterized and these same colonies were subcultured, Gram stained and submitted to catalase and oxidase tests. Bacterial isolates were subjected to automatic bacterial identification through VITEK 2 system (bioMérieux, Inc. Hazelwood, MO, USA) using commercially available identification cards for Gram-negative bacteria in accordance to the manufacturer’s recommendations. After bacterial identification, the colonies were inoculated into microtubes containing Brain-Heart Infusion (BHI) broth with 30 % glycerol and stored at - 80 °C until subsequent molecular and phenotypic tests described below.

### APEC diagnosis tests

The diagnosis of APEC strains was performed by different methods previously described. The ability of *E. coli* strains to induce lethality in 1-day-old specific-pathogen-free (SPF) chicks (detailed on section **Lethality test**) was considered gold standard test to assess strain pathogenicity. In addition, two molecular methods based on genetic profiles were used: 1) detection of minimal predictors described by Johnson et al. [7], which classify an *E. coli* strain as pathogenic based on the minimum detection of four out of five virulence genes (*iroN*, *ompT*, *hlyF*, *iss* and *iutA*); and 2) genotyping method developed by Schouler et al. [8], which is based on the identification of different associations of virulence genes (*iutA*, *sitA*, *aec26*, P (F11) fimbriae, O78, *frz*_*orf4*_) that allow the APEC strains classification in four genetic patterns of virulence (A, B, C and D).

### Serogrouping

Determination of O antigens was carried out by agglutination using antisera O1, O2, O5, O8, O15, O18, O25, O45, O78, O88, O111 and O120, according to the method described by Blanco et al. [9]. The O antisera were produced in the Laboratorio de Referencia de *Escherichia coli* (Lugo, Spain). Furthermore, PCR was performed to detect O1, O2, O4, O6, O7, O8, O12, O16, O18, O25a, O45a, O45b, O75, O78, O88 and O104 antigens, as previously described (Additional file 1: Table S1).

### Flagellar type

The strains were submitted to PCR to determine flagella type H4, H7, H8, H21 and H25 (Additional file 1: Table S1). Those strains negative for all flagellar types tested by PCR were submitted to motility test. Briefly, bacteria were grown on LB broth overnight. Then, the strains were deeply inoculated in LB plates 0.3 % agar using a Pasteur pipette and then incubated at 37 °C overnight for motility evaluation the following day [10].

### ECOR phylogenetic grouping

*E. coli* strains were classified into the four main ECOR phylogenetic groups by triplex PCR as described by Clermont et al. [11]. Strains were assigned to phylogenetic groups A, B1, B2, or D according to the amplification of the *chuA* and *yjaA* genes and the TspE4C2 fragment. Strains MG1655, ECOR26, ECOR62, and ECOR50 were used as controls for phylogenetic groups A, B1, B2, and D, respectively.

### Virulence genotyping

Total DNA extracts were prepared by a rapid boiling method [12]. The presence of genes encoding virulence factors were determined using primers and PCR amplification programs previously described, together with positive control strains (Additional file 1: Table S2).

Single PCR assays were used to detect *sfaS, focG*, *tsh*, *ibeA*, *aatA*, *neuC*, *irp2*, *ireA*, *sat*, *vat*, *astA*, *fyuA*, *hlyA*, *traT*, *cva/cvi*, *iucD*, *hra*, *iha*, *pic*, *csgA*, *tia*, *malX* (=rpai), *KpsMTII*, *cnf 1* and *cnf 2*. Furthermore, some multiplex assays were performed to detect simultaneously *clbB* and *clbN*, and *fimA*, *fim*_*avMT78*_ and *fimH*. DNA fragments were amplified in a 25-μL PCR mix containing 1 U of GoTaq®G2 Flexi DNA polymerase (Promega), 12.5 pmol of the forward and reverse primers, and 5 nmol of deoxynucleotide triphosphate mix (Eurogentec) in 1x GoTaq®G2 Flexi buffer. The PCR conditions were as follows: initial denaturation at 94 °C for 4 to 5 min, followed by 30 cycles of 94 °C for 30 s, annealing temperature according to GC-content of primers for at least 30 s, 72 °C for 30 s to 45 s according to the size of the amplified fragment (1 min/kbp), and then a final extension at 72 °C for 7 min.

### Pulsed-field gel eletrophoresis (PFGE)

Pulsed-field gel electrophoresis was conducted as previously described [13]. Bacterial cells (equivalent to an OD_600_ of 1.0) grown in BHI broth were harvested by centrifugation. The cellular pellet was resuspended in 500 μL of buffer TE 100 (10 mM Tris/HCl, pH 9, 100 mM EDTA) and incubated for 30 min at 37 °C. The bacterial suspension was mixed with an equal volume of 2.0 % low-melting-point agarose and dispensed into plug molds (Biorad). Agarose plugs were incubated in a lysozyme solution (10 mM Tris/HCl, pH 9, 100 mM EDTA, 5 mg lysozyme ml^−1^, 0.05 % sarkosyl) for 2 h at 37 °C, and then incubated overnight at 55 °C in a lysis solution (10 mM Tris/HCl, pH 9, 100 mM EDTA, 1 mg proteinase K ml^−1^, 1 % SDS). After lysis, agarose plugs were washed three times in a TE buffer (10 mM Tris/HCl, pH8, 1 mM EDTA) for 1 h at room temperature, where the first washing buffer was supplemented with 100 mM PMSF (Phenylmethylsulfonyl fluoride). For digestion, plugs were equilibrated in incubation buffer containing *XbaI* restriction enzyme (Takara) overnight. Pulsed-field gel electrophoresis was conducted in a CHEF-DRIII apparatus (Bio-Rad). Gels (1 % agarose) were run at 14 °C for 24 h in TBE buffer (4 mM Tris, 4 mM borate, 1 mM EDTA, pH 8.3) at 6 V cm^−1^. Pulse times were increased from 10 to 30 s. *XbaI* restriction fragments of *Salmonella enterica* serovar Braenderup H9812 were used as size markers. Cluster analysis using Dice similarity indices was done in BioNumerics 6.6 software (at 0.5 % tolerance and 0.5 % optimization) (Applied Maths, Ghent, Belgium) to generate a dendrogram describing the relationships among PFGE profiles.

### Multilocus Sequence Typing (MLST)

The phylogenetic relationships between strains were studied using MLST method initially described by Maiden et al. [14] and *E. coli* Achtman’s scheme (http://mlst.warwick.ac.uk/mlst/dbs/Ecoli/). *E. coli* MLST scheme used internal fragments of seven house-keeping genes: *adk* (adenylate kinase), *fumC* (fumarate hydratase), *gyrB* (DNA gyrase), *icd* (isocitrate/isopropylmalate dehydrogenase), *mdh* (malate dehydrogenase), *purA* (adenylosuccinate dehydrogenase) and *recA* (ATP/GTP binding motif). They were amplified in a total volume of 20 μL containing 4 μL of DNA crude extract as a template, 2.5 U of GoTaq®G2 Flexi DNA polymerase (Promega), 10 pmol of each primer, 5 nmol of deoxynucleoside triphosphate 30 mM MgCl_2_ in 1x buffer. PCR conditions were as follows: 94 °C for 5 min; 30 cycles of 94 °C for 40 s, variable annealing temperature (54 °C, 60 °C, 64 °C, 58 °C, 62 °C, 62 °C or 58 °C, respectively) for 45 s, and 72 °C for 45 s; and a final extension at 72 °C for 5 min. The amplicons were sequenced on both strands and sequence type (ST) of each allele was attributed according to Achtman’s scheme. Novel STs described in this work were submitted to the *E. coli* MLST database and identified as ST5856 and ST5766.

### Lethality test

Strain virulence was evaluated by a lethality test using 1-day-old chicks as previously described [15]. Lethality test was carried out in the experimental infection unit PFIE (Plateforme d’Infectiologie Expérimentale, INRA Val de Loire). For each strain, groups of five 1-day-old SPF chicks were inoculated subcutaneously with 0.5 mL of an overnight culture in LB-Miller broth without agitation (inoculum in stationary phase was ~10^8^ CFU). Mortality was recorded at 4 days post inoculation and the strains were classified as pathogenic when at least one chick died [16]. Avian *E. coli* strains BEN2908 and BEN2269 (a non-pathogenic avian *E. coli* isolate of serogroup O2) were used as positive (5 chicks died) and negative control (no chicks died), respectively. The housing, husbandry and slaughtering conditions conformed to European Guidelines for care and use of laboratory animals. French regional ethics committee number 19 (Comité d’Ethique en Expérimentation Animale Val de Loire) approved this protocol under the reference 2012-11-5.

### Serum bactericidal test

The serum bactericidal assay was performed as previously described by Dozois et al. [17] with some modifications. Briefly, bacteria were grown overnight in LB broth at 41 °C with agitation (180 rpm). Then, bacterial cultures were resuspended in fresh medium (OD_600_ = 0.02), incubated at 41 °C with agitation (180 rpm), and harvested during the logarithmic growth phase (DO_600_ = 0.35). Bacteria were washed at room temperature with dulbeco’s phosphate-buffered saline (pH 7.0 ~ 7.3) and then resuspended to a concentration of 2x10^6^ CFU/mL. A volume of 500 μL of bacterial suspension was added to 500 μL of complement or inactivated (56 °C, 30 min) SPF chicken serum, which were incubated at 41 °C without agitation. Viable cell counts were counted at 0 h and 3 h by plating 10-fold dilutions in sterile saline solution on LB agar plates. Compared to the bacterial count in inactivated serum, a strain was considered resistant when the bacterial count increased or did not change, intermediate when the bacterial count decreased up to one order of magnitude, and sensitive when bacterial count decreased more than one order of magnitude. Serum resistant (BEN2908) and serum sensitive (BEN4134) *E. coli* strains were used as positive and negative controls.

### Antibiotic susceptibility testing

Susceptibility testing was performed by the disk diffusion method according to the guidelines of the Antibiogram Committee of the French Society of Microbiology (http://www.sfm-microbiologie.org). The antibiotics tested belong to seven different classes: aminoglycosides (gentamicin, Gen; neomycin, Neo; apramycin, Apr), beta-lactams (amoxicillin, Amx; amoxicillin + clavulanic acid, Amc), cephalosporins (cephalotin, Cef; cefoxitin, Fox; ceftiofur, Xnl), phenicols (florfenicol, Ffc), polypeptides (colistin, Cst), quinolones (nalidixic acid, Nal; flumequine, UB; enrofloxacin, Enr), sulfonamides (trimethoprim, Tmp; Tmp + sulfamethoxazole, TmpStx), and tetracyclines (tetracycline, Tet). The presence of extended spectrum β-lactamases (ESBL) was detected by double-disk synergy method [18]. *E. coli* ATCC 25922 strain was used as quality control.

## Results

### Epidemiological features of *E. coli* strains and PFGE

*E. coli* strains were isolated from eight flocks in the municipalities of Belo Horizonte, Bom Jesus de Amparo, Igarapé, Igaratinga, Itabira and São Sebastião do Oeste, all located in the state of Minas Gerais, Brazil. Management and biosecurity practices varied among the farms, with broilers number per flock ranging from 20,000 to 40,000. Broiler farms usually raise broilers up to approximately 42 to 45 days before processing them. Broilers studied were 40 to 56 days-old (average of 46 days-old). Antibiotics usage in sampled farms included enrofloxacin, fosfomycin, amoxicillin, and trimethoprim sulfa, which were most commonly used to treat respiratory or enteric diseases. Antibiotics such as zinc bacitracin and colistin were confirmed to be frequently used as growth promoters, although information on its use was not available for all farms.

**Fig. 1:**
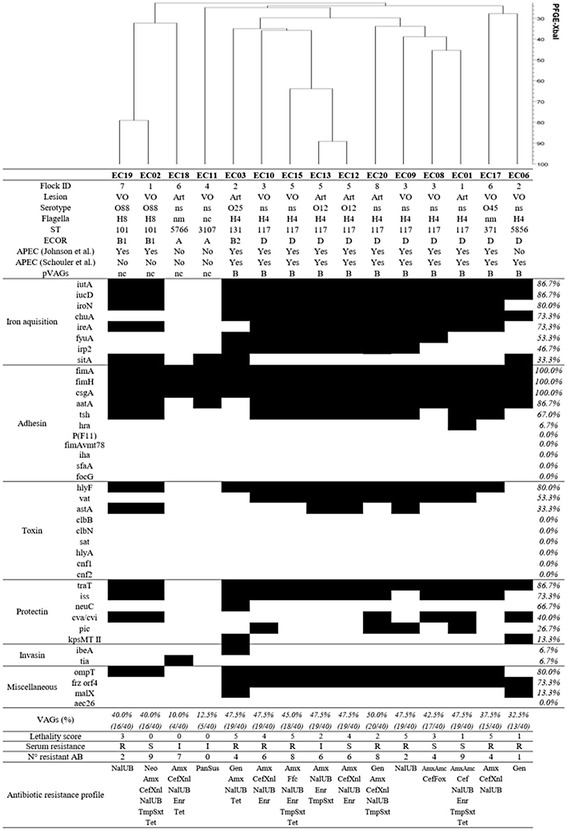
Molecular and phenotypic characterization of 15 *Escherichia coli* strains isolated from broilers with osteomyelitis and arthritis. Black and white boxes represent positive and negative results, respectively. *Flock ID*, number of the flock of origin; *Lesion, VO*: vertebral osteomyelitis, *Art*: arthritis; *Serotype*, *ns*: non-serotyped; *Flagella*, *nm*: non-motile, *nc*: non-correspondent to any flagellar type tested; *ST*, Sequence type; *ECOR*: ECOR phylogenetic group; *APEC (Johnson* et al.*)*: APEC diagnosis according to Johnson et al. (2008); *APEC (Schouler* et al.*)*; APEC diagnosis according to Schouler et al. (2012); *Yes*: APEC strain, *No*: non-APEC strain; *pVAGs*, pattern of virulence genes described by Schouler et al. (2012), *nc*: non-correspondent to the described patterns; *Iron acquisition*, genes encoding iron acquisition system; *Adhesin*, genes encoding adhesins; *Toxin*, genes encoding toxins; *Protectin*, genes encoding protectins; *Invasin,* genes encoding invasins; *Miscellaneous*, genes encoding different kinds of virulence; *VAGs (%)*, percentage of APEC-associated virulence genes; *Lethality score,* number of chicks that died at the fourth day post-infection with *E. coli*; *Serum resistance*, *R*: serum resistant strain, *I*: intermediate resistant strain, *S*: serum sensitive strain; *N° resistant AB*: number of antibiotics to which the strain was resistant; *Antibiotic resistance profile*: gentamicin, Gen; neomycin, Neo; apramycin, Apr; amoxicillin, Amx; amoxicillin + clavulanic acid, Amc; cephalotin, Cef; cefoxitin, Fox; ceftiofur, Xnl; florfenicol, Ffc; colistin, Cst; nalidixic acid, Nal; flumequine, UB; enrofloxacin, Enr; trimethoprim, Tmp; Tmp + sulfamethoxazole, TmpStx; tetracycline, Tet; pansusceptible, PanSus

The fifteen *E. coli* strains presented different genetic profiles and revealed to be highly diverse, even for same flock isolates (Fig. 1).

### Clinic and pathological findings of the diseases

#### Vertebral osteomyelitis

**Fig. 2:**
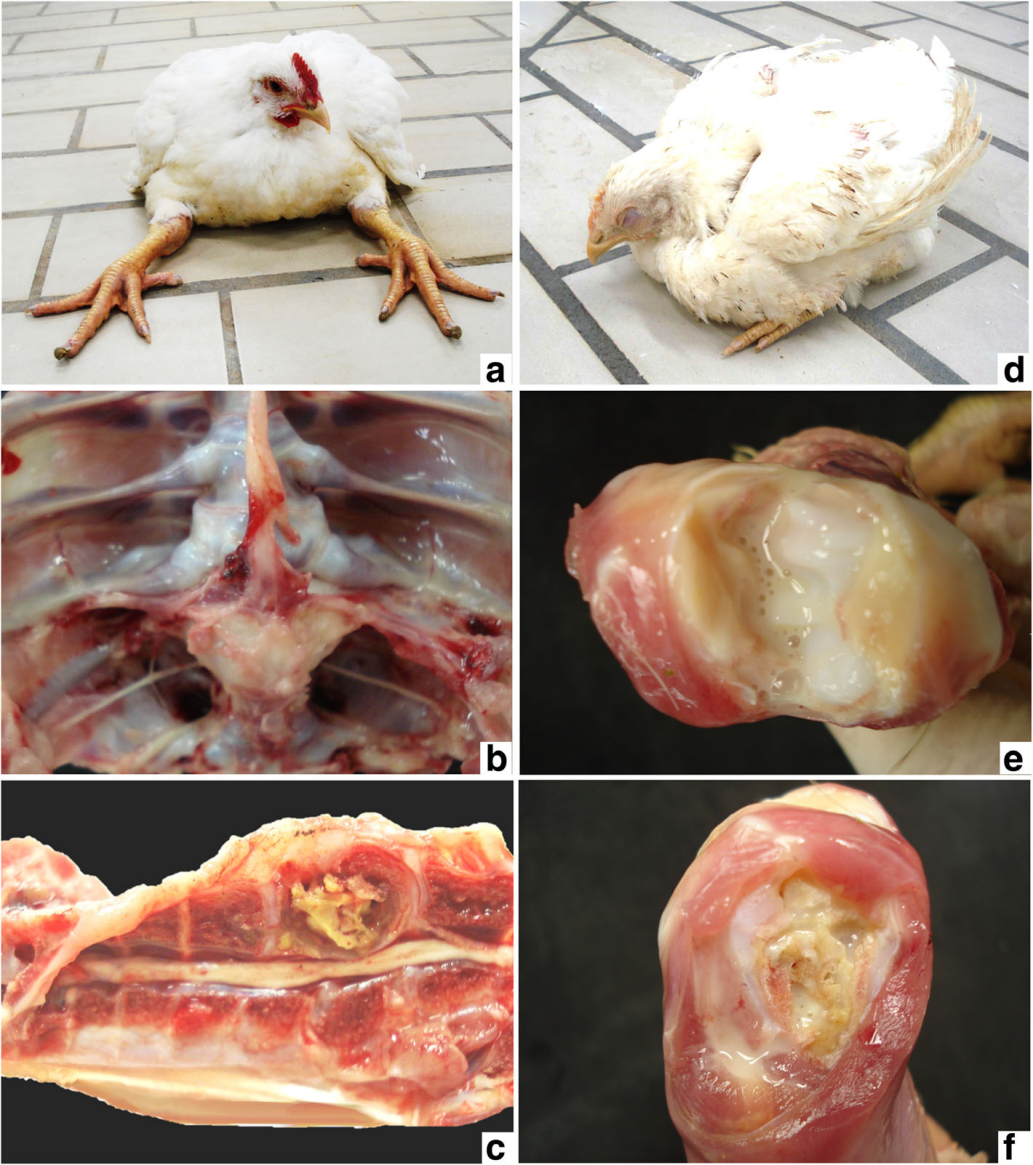
Clinical signs and gross pathology of vertebral osteomyelitis (**a**, **b**, **c**) and arthritis (**d**, **e**, **f**) in broilers. **a** Broiler showing the classical clinical sign of severe cases of vertebral osteomyelitis. **b** Note the enlargement of affected vertebral body (T4), **c** which revels caseonecrotic material and spinal cord compression on longitudinal section. **d** Broiler with bilateral arthritis showing ventral recumbency and retracted legs. **e** Suppurative exudate in articular cavity in acute arthritis, **f** which extended to proximal tibiotarsus causing tibial osteomyelitis

**Fig. 3:**
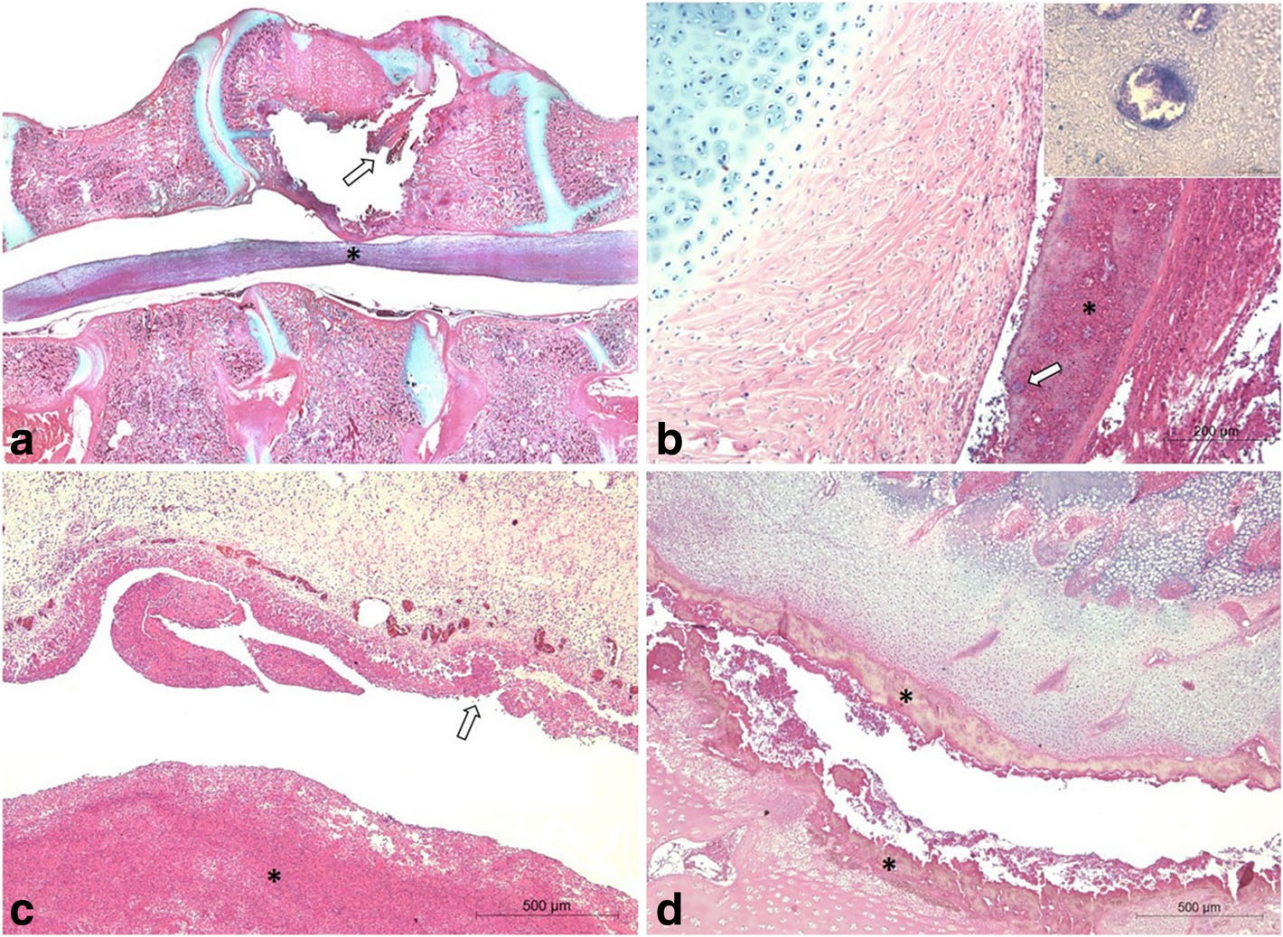
Histopathology of osteomyelitis and arthritis in broilers. **a** Vertebral osteomyelitis showing enlargment of vertebral body (T4) by caseonecrotic material (remanescent, arrow), which compresses spinal cord (*); HE. **b** Caseonecrotic hererophilic and histiocytic exudate (*) in the articular space with intralesional bacterial colonies (arrow); HE. *Inset*: Gram-negative bacteria stained by Goodpasture. **c** Necrotic synovitis (arrow) associated with caseonecrotic exudate within the articular space (*); HE. **d** Proximal growth plate (physis) of tibiotarsus showing extensive necrosis (*) with heterophilic exudate in a case of tibial osteomyelitis; HE

The clinic and pathological findings and the total of broilers examined were previously described in details by Braga et al. [6]. Clinically, affected broilers presented partial or total gait impairment according to the degree of vertebral body enlargement (T4 vertebra) and consequent spinal cord compression, which varied from mild to severe (Fig. 2a). Gross lesions were characterized by caseonecrotic osteomyelitis with protrusion of affected vertebral body and spinal cord compression (Fig. 2b, c). Histopathological evaluation of affected vertebral body included caseonecrotic osteomyelitis frequently associated with intralesional Gram-negative bacteria, besides degeneration and necrosis of overlying spinal cord (Fig. 3a).

### Arthritis

Broilers presented different degrees of limited mobility depending on the joint lesion site (unilateral or bilateral). When there was involvement of only one leg, broilers could stay in station, although avoiding to support the affected limb on the floor. In bilateral cases, birds often remained in ventral recumbency, with retracted members and supporting their weight on the pectoral muscles (Fig. 2d). Gross evaluation showed swollen of affected joints and, on cut surface, the aspect of lesions varied according to the course of disease. In acute lesions, there was mild to moderate suppurative exudate within synovial fluid and involving articular capsule, occasionally extending to adjacent tendon sheaths, musculature, and subcutaneous tissue (Fig. 2e). In one case, the inflammatory process extended to adjacent proximal tibiotarsus leading to tibial osteomyelitis characterized by indistinct growth plate and metaphysis, which were replaced by necrosuppurative exudate (Fig. 2f). In chronic cases, there was moderate to severe caseofibrinous arthritis. Occasionally, acute arthritis in an antimere and chronic arthritis in the contralateral antimere were observed in the same broiler. Histopathological analysis of acute lesions revealed moderate to intense fibrinoheterophilic and histiocytic arthritis. In chronic cases, there was caseonecrotic heterophilic and histiocytic arthritis, often associated with myriads of intralesional bacterial colonies (Fig. 3b). Furthermore, necrotic synovitis and synovial hyperplasia were occasionally observed (Fig. 3c). In lesions with greater intensity and extension, involvement of adjacent periarticular structures was characterized by degeneration and necrosis of skeletal muscles or osseous tissue (Fig. 3d) associated with hyperemia, infiltration of heterophils and macrophages and fibrin. In addition, proliferation of fibrous tissue in the articular capsule and adjacent tissue was found in more advanced cases.

### APEC diagnosis

According to lethality test, 12 *E. coli* strains were considered as APEC, since four strains killed 5 out 5 chicks, two strains killed 4 out 5 chicks, two killed 3 out 5 chicks, two killed 2 out 5 chicks, and two killed 1 out 5 chicks. One strain (EC02) did not kill any chick, but was considered as APEC according to Johnson et al. [7], which classify an *E. coli* strain as pathogenic based on the presence of minimum four out of five virulence genes carried by plasmids associated with highly pathogenic APEC. The results of molecular tests previously described to diagnose APEC showed an agreement of 80.0 % (12/15) (Fig. 1). According to Johnson et al. [7] and Schouler et al. [8], 10 *E. coli* strains were considered APEC and two were considered as non-pathogenic strains. Although there were three discrepancies between both tests, these three APEC strains were diagnosed alternatively by one of the tests and the final criteria for APEC diagnosis was the lethality test.

### Group O serotyping and flagella

Different serogroups were detected among the strains, mainly O12 (13.3 %), O88 (13.3 %), O25 (6.7 %), and O45 (6.7 %) (Fig. 1). High percentage (60.0 %) of strains did not correspond to any of the O somatic antigen surveyed in this study, and were classified as non-serotyped (NS). The most prevalent flagellar types were H4 (66.7 %) and H8 (13.3 %), and only for one motile strain it was not possible to identify the corresponding flagella (Fig. 1). H4 was detected in O12, O25 and non-serotyped strains, while O88 strains were H8.

### MLST and ECOR phylogroups

The strains were assigned to seven different sequence types (STs) (Fig. 1). Most strains (86.7 %, 13/15) were grouped in known STs, while 13.3 % (2/15) were new STs described in this work. The most frequent ST was ST117 and represented 53.3 % (8/15) of *E. coli* strains, followed by ST101 (13.3 %, 2/15). ST131, ST371 and ST 3107 were identified once each. When classified into ECOR phylogenic groups, most strains were D (66.7 %, 10/15), followed by A (13.3 % 2/15), B1 (13.3 %, 2/15) and B2 (6.7 %, 1/15) (Fig. 1).

### Virulence genes profile

APEC strains showed highly variable content of virulence genes, although those responsible for iron acquisition and adhesion were detected more frequently (Fig. 1). The non-pathogenic strains showed marked lack of virulence genes when compared to APEC strains, with higher content of adhesin encoding genes.

### Bactericidal effect of serum

High percentage of *E. coli* strains, 53.3 % (8/15), was serum resistant, while 33.4 % (5/15) was characterized as serum sensitive and 13.3 % (2/15) as intermediate strains (Additional file 1: Figure S1).

### Antibiotic resistance profile

**Fig. 4:**
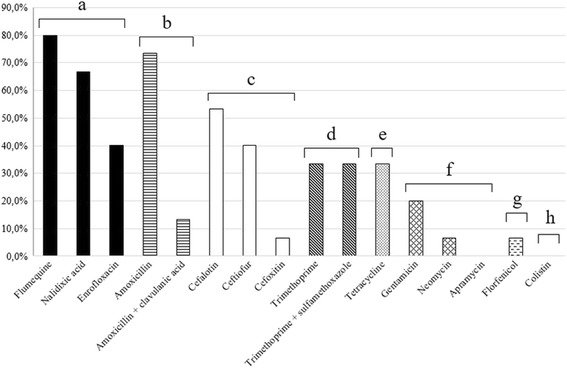
Percentages of antibiotic resistance of *E. coli* strains isolated from vertebral osteomyelitis and arthritis in broilers by antibiotic class: (**a**) quinolones; (**b**) beta-lactams; (**c**) cephalosporins; (**d**) sulfonamides; (**e**) tetracyclines; (**f**) aminoglycosides; (**g**) phenicols; and (**h**) polypeptides

The *E. coli* strains studied presented a large diversity of antibiotic resistance profiles (Fig. 1). One *E. coli* strain was pansusceptible, but high percentage (73.0 %) of strains were resistant to more than three classes of antibiotics and defined as multidrug-resistant *E. coli*. These eleven multidrug-resistance strains were mainly characterized by resistance to amoxicillin (100.0 %), enrofloxacin (54.5 %), ceftiofur (54.5 %), and tetracycline (45.4 %). The *E. coli* strains were more resistant to nalidixic acid (quinolone class) and amoxicillin (beta-lactam class), 80.0 % and 73.3 % respectively (Fig. 4). Susceptibility or low resistance to polypeptides (0.0 %,) and phenicols (6.7 %,) were observed. One non-pathogenic *E. coli* strain (EC18) was suspected to produce an ESBL by the synergy observed between amoxicillin/clavulanic acid and ceftiofur using the disk diffusion method.

## Discussion

Our results showed that *E. coli* strains involved in vertebral osteomyelitis and arthritis cases in broilers in Brazil are highly diverse. We observed that the same disease (i.e., vertebral osteomyelitis) was caused by genetically diverse *E. coli* strains with different pathogenicity traits in the same flock (flock 3). Furthermore, genetically diverse strains were recovered from different diseases (i.e., vertebral osteomyelitis or arthritis) in the same flock (flocks 2, 5 and 6). These findings show that both diseases are not caused by a unique *E. coli* strain. Other authors also report genetic diverse populations of *E. coli* in field cases of colibacillosis in a single flock [19] or in different flocks [20].

*E. coli* is one of the bacteria described as etiological agent of vertebral osteomyelitis [5]. Recent data on etiological agents of this disease in broilers described involvement of single or mixed bacteria including *Enterococcus* spp., *E. faecalis*, *E. hirae* and *Staphylococcus aureus*, besides *E. coli* [6]*.* This feature can be similar to what have been previously proposed on turkey osteomyelitis complex (TOC), in which bacterial arthritis and osteomyelitis are associated to involvement of many different opportunistic microorganisms, suggesting that is likely to be influenced by factors such as immunosuppression rather than by the pathogenicity intensity of these bacteria [21].

Diversity in serogroups among *E. coli* strains was also remarkable, as exemplified by the detection of serogroup O12, which up to now was not described in Brazilian *E. coli* strains from human or animal origin. Strains belonging to this serogroup exhibited profile O12:H4-ST117 and were isolated from two broilers from the same flock presenting only arthritis. Previous studies on serogroup determination of *E. coli* isolated from septicemic and healthy broilers revealed that O12 was involved in only 1 % of colisepticemia cases, but was one of the serogroups predominantly identified among septicemic *E. coli* [22]. O12 *E. coli* strain was also reported in human, isolated from an immunocompetent woman with a history of repeated amnion infections and spontaneous abortion [23].

An *E. coli* strain O45:HNM-D-ST371 was also detected in this study. This type of strain has been described previously in 16.4 % (9/55) of O45 *E. coli* strains isolated from avian colibacillosis cases in Europe and it was identified only in O45 *E. coli* strains of avian origin, different from O45:K1:H7-B2-ST95 identified in avian and human *E. coli* isolates [24]. However, this last one was not detected in Brazilian APEC strains described here and in previous studies [25].

ST 117, which represents more than half of our *E. coli* strains, and ST 131 were involved in osteomyelitis and arthritis cases. These STs are commonly shared by APEC and human ExPEC strains [25]. Close genetic relations have been detected in ST 117 *E. coli* strains of animal and human origin, which have been identified in large poultry producers such as Brazil [25], USA [26], and also Egypt [27], Denmark [28], Sri Lanka [29], and South Korea [30].

We also identified two ST101 APEC strains, which belonged to phylogroup B1, serotype O88:H8 and were non-ESBL as evaluated by the disk diffusion method. This ST was not related to infections caused by APEC until recently, when one O15:H10-B1-ST101 APEC strain was isolated from colibacillosis associated lesions in Spain [20].

ST131 is a globally disseminated multidrug resistance clone, responsible for urinary tract and bloodstream infections in humans. Its rapid emergence and successful spread is strongly associated with antibiotic resistance [31–33]. One O25:H4-B2-ST131 *E. coli* strain was detected in a broiler joint with arthritis in this study. In Brazil, this clone was previously detected in APEC strains recovered from broilers with different visceral lesions [34] and from APEC and human ExPEC collections [25]. Although few data are available on this clonal group from poultry, Mora et al. [35] reported an increasing presence of clonal group O25b:H4-ST131 in retail chickens. Interestingly, a retail chicken sample revealed macrorestriction profile indistinguishable from an *E. coli* strain isolated from a human with urinary tract infection [36].

High percentage of multidrug resistant *E. coli* was detected in this study. It is known that *E. coli* strains isolated from poultry frequently show resistance to more than one antimicrobial drug [37], which represents a global concern. It has been shown that poultry workers may have increased risk of carrying multidrug-resistant *E. coli*, which demonstrates that occupational exposure to antimicrobial-resistant *E. coli* from live-animal contact in the broiler industry may be an important route of entry for antimicrobial-resistant *E. coli* into the community [38].

Most *E. coli* strains analyzed in this study exhibited resistance to at least one antibiotic from different main classes: beta-lactams, cephalosporins and quinolones. Resistance to these antibiotic classes is a chronic problem described for avian *E. coli* strains isolated in Brazil [34, 39]. A concern is the increasing resistance to ceftiofur, which was evident when we compared our *E. coli* strains to those isolated from broilers in previous years in Brazil [34]. This finding is probably the result of increasing usage of this drug in poultry and highlights the need for responsible and controlled use of antibiotics in animals. A major public health concern is that the use of third-generation cephalosporins, such as ceftiofur, in food animals is leading to resistance to other extended-spectrum cephalosporin molecules, which are used in the treatment of many different human infections [40].

Tetracycline resistance level of the *E. coli* strains studied was lower than that described in other regions of Brazil [34, 39] and in other countries, such as China, where resistance to tetracycline can reach about 90 % [41]. For many years, tetracycline was used as prevention and as growth promoter in poultry, but the use of antibiotics with these purposes was banned since 2009 in Brazil. In the state of Minas Gerais, where samples were collected, tetracycline use has no longer being recommended by poultry veterinarians due to its prohibition and bacterial resistance (personal information). These data suggest that the discontinued use of tetracycline in poultry in the region may have provided an increase in the number of *E. coli* strains susceptible to this drug, as described for *Salmonella* strains in USA, where it was observed significant reduction of humans and swine strains resistant to tetracycline after its prohibition as prophylactic drug in animal feed [42].

All 15 *E. coli* strains studied were isolated from the exudate of osteomyelitis or arthritis lesions. The strains EC11 and EC18 classified as non-pathogenic were also isolated from broilers with vertebral osteomyelitis and arthritis, respectively. In these cases, necrotic and inflammatory lesions were associated with bacterial colonies constituted by Gram negative rods, including strains classified as non-APEC. The single or double colonies picked up from the pure culture of *E. coli* probably resulted in the selection of a non-APEC clone, once it is known that in the same lesion it is possible to find distinct *E. coli* clone populations. In order to avoid the selection of a non-representative bacterial clone, it is recommended to select and mix several colonies from the pure culture isolated from the lesion for further evaluation [43]. This procedure provides more efficient results, especially regarding to antimicrobial susceptibility, since it can reduce a possible variation in the susceptibility of isolated clones and improve the selection of antibiotics for treatment.

The broilers had no additional gross lesions in other sites at necropsy, except in two: one exhibited vertebral osteomyelitis and intertarsal arthritis and another broiler had intertarsal arthritis with osteomyelitis in proximal tibiotarsus of the same antimere. Although the information on previous respiratory disease was not available for all flocks studied, it is known that colibacillosis is frequent in broilers of the region (laboratory and field observations). Localization of *E. coli* in bones and synovial tissues is a common sequel of colisepticemia [3]. Some studies with turkeys demonstrated that often multiple sites are involved and the bones most often affected are tibiotarsus, femur, thoracolumbar vertebra, and humerus [44]. Bacteria colonizing the vascular sprouts that invade the physis of a growing bone, provoke an inflammatory response that results in osteomyelitis. Transphyseal blood vessels in birds serve as conduits for the process to spread bacteria into the joint and surrounding soft tissues [45].

## Conclusions

Our results showed that highly diverse *E. coli* strains can be recovered from osteomyelitis and arthritis in broilers, even in the same flock. Based on molecular and phenotypic characteristics, there is no specific virulence pattern of the *E. coli* associated with vertebral osteomyelitis or arthritis. Some of the strains involved in these diseases are belonged to STs commonly shared by animals and humans, similar to others previously isolated from different lesions in broilers. Most of these strains are multidrug resistant, with increasing rates of ceftiofur resistance, which is a public and animal health concern. These findings highlight the importance of appropriate management practices, which are valuable in preventing and controlling colibacillosis, thus reducing the need for antibiotics use in animals.

## Additional file

Additional file 1: Table S1. Serogrouping and Flagellar type and primers used for PCR amplification. **Table S2.** Virulence genotyping and primers used for PCR amplification. **Figure S1.** Serum resistance of *E. coli* strains in complete SPF chicken serum (a) and inactivated SPF chicken serum (b). (DOC 571 kb)
