# Supplementary material for: Diversity of Escherichia coli strains involved in vertebral osteomyelitis and arthritis in broilers in Brazil
Source: BMC Vet Res. 2016 Jul 14;12:140. doi: 10.1186/s12917-016-0762-0 (PMC5477814; doi:10.1186/s12917-016-0762-0)
Supplement: Additional file 1: Table S1. — Serogrouping and Flagellar type and primers used for PCR amplification. Table S2. Virulence genotyping and primers used for PCR amplification. Figure S1. Serum resistance of E. coli strains in complete SPF chicken serum (a) and inactivated SPF chicken serum (b). (DOC 571 kb) [file 12917_2016_762_MOESM1_ESM.doc]

**SUPPLEMENTARY DATA**

**Methods**

**Serogrouping and Flagellar type**

Table 1

| *Target* | *Primer* | *Sequence (5’-3’)* | *Fragment size (bp)* | *TM (ºC)* | *(+) Control* | *Reference* |
| --- | --- | --- | --- | --- | --- | --- |
| Serogroup O | gndbis.f | ATACCGACGACGCCGATCTG | - | - | - | Clermont et al. [1] |
| Serogroup O1 | rfbO1.r | CCAGAAATACACTTGGAGAC | 189 | 56 | BEN1438 |  |
| Serogroup O6 | rfbO6a.r | AAATGAGCGCCCACCATTAC | 584 | 59 | BEN2936 |  |
| Serogroup O7 | rfbO7.r | CGAAGATCATCCACGATCCG | 722 | 59 | BEN2845 |  |
| Serogroup O12 | rfbO12.r | GTGTCAAATGCCTGTCACCG | 239 | 59 | BEN355 |  |
| Serogroup O16 | rfbO16.r | GGATCATTTATGCTGGTACG | 450 | 59 | BEN2198 |  |
| Serogroup O18 | rfbO18.r | GAAGATGGCTATAATGGTTG | 360 | 59 | BEN2744 |  |
| Serogroup O25a | rfbO25a.r | GAGATCCAAAAACAGTTTGTG | 313 | 59 | ECOR51 |  |
| Serogroup O45a | rfbO45a.r | GCGCAATAAATGGCTGACTG | 312 | 58 | BEN4190 |  |
| Serogroup O45b | rfbO45b.r | TGCGAGTAGACTATCTCAAG | 436 | 58 | BEN5054 |  |
| Serogroup O75 | rfbO75.r | GTAATAATGCTTGCGAAACC | 419 | 59 | ECOR64 |  |
| Serogroup O88 | rfbO88.r | AAGGAAAAACGCTGGGAGAG | 494 | 55 | ECOR26 | Clermont et al. [2] |
| Serogroup O104 | rfbO104.r | TGGCTTAGGATACTTGCAGC | 410 | 52 | BEN4438 | Clermont et al. [1] |
| Serogroup O2 | wzyO2-F | TGCAACTCATTGGTCTGCTTTGCC | 351 | 56 | ECOR62 | Fratamico et al. [3] |
|  | wzyO2-R | CGGAAAGCCATAACAGGTAGAGAG |  |  |  |  |
| Serogroup O4 | wzxO4-F | TTGTTGCGATAATGTGCATGTTCC | 664 | 58 | ECOR66 | Li et al. [4] |
|  | wzxO4-R | AATAATTTGCTATACCCACACCCTC |  |  |  |  |
| Serogroup O8 | O8-F | CCAGAGGCATAATCAGAAATAACAG | 448 | 55 | BEN352 | Li et al. [4] |
|  | O8-R | GCAGAGTTAGTCAACAAAAGGTCAG |  |  |  |  |
| Serogroup O78 | AT7 | GGTATCGGTTTGGTGGTA | 992 | 52 | ECOR70 | Liu et al. [5] |
|  | AT8 | AGAATCACAACTCTCGGCA |  |  |  |  |
| Flagella H4 | fliC-H4-F | GGCGAAACTGACGGCTGCTG | 201 | 66 | BEN4185 | Bielaszewska et al. [6] |
|  | fliC-H4-R | GCACCAACAGTTACCGCCGC |  |  |  |  |
| Flagella H7 | fliC-H7f | CCACGACAGGTCTTTATGATCTGA | 96 | 58 | BEN4190 | Bugarel et al. [7] |
|  | fliC-H7r | CAACTGTGACTTTATCGCCATTCC |  |  |  |  |
| Flagella H8 | fliC-H8f | AAAGGCTCCATTGAATACAAGG | 108 | 62 | BEN4198 |  |
|  | fliC-H8r | TTGACCATCAATATTTGCGGTC |  |  |  |  |
| Flagella H21 | fliC-H21f | TACTAGTGCAACCGTTGCC | 102 | 58 | BEN4197 |  |
|  | fliC-H21r | AGATCAGATAGTGTCGCTGC |  |  |  |  |
| Flagela H25 | fliC univ-F | ATGGCACAAGTCATTAATAC | 559 | 57 | BEN1424 | Iguchi et al. [8] |
|  | fliC-H25-R | TGCGGGATAGATGTGATAGCA |  |  |  |  |

Primers used for PCR amplification

**Virulence genotyping**

Table 2

| *Gene* | | *Primer* | *Sequence (5´-3´)* | *TM (°C)* | *Product (bp)* | *(+) Control* | *Reference* |
| --- | --- | --- | --- | --- | --- | --- | --- |
| *aatA* | APEC autotransporter gene | aatA-F  aatA-R | ATGAATAAGAATATACGAATTTTAC  ACCATTATTATTTAGCGTAAAG | 52 | 300 | BEN194 | Dai et al. [9] |
| *aec26* | Avian *E. coli* gene 26 (=A9) | aec26-F  aec26-R | ATGAGCGATATGAGTGAAGC  TTATCGGAGTAATTTATTGA | 53 | 760 | BEN2908 | Schouler et al. [10] |
| *astA* | Aggregative stable enterotoxin | astA-F  astA-R | TGCCATCAACACAGTATATC  TCAGGTCGCGAGTGACGG | 58 | 116 | BEN194 | Yamamoto and Nakazawa [11] |
| *chuA* | Heme binding protein | chuA.1  chuA.2 | GACGAACCAACGGTCAGGAT  TGCCGCCAGTACCAAAGACA | 59 | 279 | BEN2908 | Clermont et al. [12] |
| *clbB* | Colibactin polyketide synthesis system | clbB-F  clbB-R | GATTTGGATACTGGCGATAACCG  CCATTTCCCGTTTGAGCACAC | 62 | 579 | BEN2742 | Johnson et al. [13] |
| *clbN* | Colibactin polyketide synthesis system | clbN-F  clbN-R | GTTTTGCTCGCCAGATAGTCATTC  CAGTTCGGGTATGTGTGGAAGG | 62 | 733 | BEN2742 | Johnson et al. [13] |
| *cnf1* | Cytotoxic necrotizing factor type 1 | cnf1-A  cnf1-B | GAACTTATTAAGGATAGT  CATTATTTATAACGCTG | 50 | 543 | BEN2987 | Blanco et al. [14] |
| *cnf2* | Cytotoxic necrotizing factor type 2 | cnf2-F  cnf2-R | AATCTAATTAAAGAGAAC  CATGCTTTGTATATCTA | 48 | 543 | BEN2340 | Blanco et al. [14] |
| *csgA* | Structural subunit of the curli fimbriae | csgA-F  csgA-R | AGAGACAGTCGCAAATGGCTA  AGTACTGATGAGCGGTCGCGT | 55 | 538 | BEN2936 | This work |
| *cva/cvi* | Strutural genes of colicin V operon | cva/cvi-F  cva/cvi-R | TCCAAGCGGACCCCTTATAG  CGCAGCATAGTTCCATGCT | 60 | 598 | BEN2908 | Ewers et al. [15] |
| *fimA* | Major type 1 subunit fimbriae (pilin) | fimA1  fimA2 | CGGCTCTGTCCCTSAGT  GTCGCATCCGCATTAGC | 52 | 500 | BEN2908 | Moulin-Schouleur et al. [16] |
| *fim_avMT78_* | fimA variant of MT78 | fimA201  fimA215 | TCTGGCTGATACTACACC  ACTTTAGGATGAGTACTG | 52 | 266 | BEN2908 | Marc and Dho-Moulin [17] |
| *fimH* | Minor fimbrial subunit, D-mannose specific adhesin | fimH2  fimH17 | GATCTTTCGACGCAAATC  CGAGCAGAAACATCGCAG | 52 | 389 | BEN2908 | Arné et al. [18] |
| *focG* | G adhesin of the type F1C fimbriae | focG-F  focG-R | CAGCACAGGCAGTGGATACGA  GAATGTCGCCTGCCCATTGCT | 63 | 362 | BEN2936 | Johnson and Stell [19] |
| *frz_orf4_* | Sugar metabolism (=D7) | frz-F  frz-R | TCAGTAAGAACGAAAGTGTG  ACAGGAACAATCCCGTGGAT | 53 | 565 | BEN2908 | Moulin-Schouler et al. [16] |
| *fyuA* | Ferric yersinia uptake | fyuA-F  fyuA-R | GCGACGGGAAGCGATGACTTA  CGCAGTAGGCACGATGTTGTA | 64 | 774 | BEN2908 | Schubert et al. [20] |
| *hlyA* | Hemolysin A | hlyA-F  hlyA-R | GTCCATTGCCGATAAGTTT  AAGTAATTTTTGCCGTGTTTT | 50 | 351 | J96 | Ewers et al. [21] |
| *hlyF* | Putative avian hemolysin | *hlyF-F*  *hlyF-R* | GGCCACAGTCGTTTAGGGTGCTTACC  GGCGGTTTAGGCATTCCGATACTCAG | 63 | 450 | BEN2908 | Johnson et al. [13] |
| *hra* | Heat-resistant agglutinin | hra-F  hra-R | GTAACTCACACTGCTGTCACCT  CGAATCGTTGTCACGTTCAG | 62 | 139 | BEN2908 | Ewers et al. [15] |
| *ibeA* | Invasion brain endothelium | ibeA-F  ibeA-R | TGAACGTTTCGGTTGTTTTG  TGTTCAAATCCTGGCTGGAA | 55 | 814 | BEN2908 | Germon et al. [22] |
| *iha* | Bifunctional enterobactin receptor/adhesin protein | iha-F  iha-R | TAGTGCGTTGGGTTATCGCTC  AAGCCAGAGTGGTTATTCGC | 60 | 609 | BEN2936 | Ewers et al. [15] |
| *ireA* | Iron-responsive element | ireA-F  ireA-R | ATTGCCGTGATGTGTTCTGC  CACGGATCACTTCAATGCGT | 60 | 385 | BEN2936 | Ewers et al. [15] |
| *iroN* | Salmochelin siderophore receptor gene | *iroN-F*  *iroN-R* | AATCCGGCAAAGAGACGAACCGCCT  GTTCGGGCAACCCCTGCTTTGACTTT | 63 | 553 | BEN2908 | Johnson et al. [13] |
| *irp2* | Iron-repressible protein | irp2-F  irp2-R | AGGATTCGCTGTTACCGGAC  TCGTCGGGCAGCGTTTCTTCT | 62 | 286 | BEN2908 | Schubert et al. [20] |
| *iss* | Episomal increased serum survival gene | *iss-F*  *iss-R* | CAGCAACCCGAACCACTTGATG  AGCATTGCCAGAGCGGCAGAA | 63 | 323 | BEN2908 | Johnson et al. [13] |
| *iucD* | Aerobactin synthesis | iucD-F  iucD-R | CCTGATCCAGATGATGCTC  CTGGATGAGCAGAAAATGACA | 56 | 193 | BEN2908 | Frömmel et al. [23] |
| *iutA* | Aerobactin siderophore receptor | iutA1  iutA15 | ATGAGCATATCTCCGGACG  CAGGTCGAAGAACATCTGG | 56 | 587 | BEN2908 | Moulin-Schouler et al. [16] |
| *kpsMT II* | Group II capsule polysaccharide synthesis | kpsMTII-F  kpsMTII-R | GCGCATTTGCTGATACTGTTG  CATCCAGACGATAAGCATGAGCA | 63 | 272 | BEN2936 | Johnson and Stell [19] |
| *malX (=rpai)* | Pathogenicity-associated island marker CFT 073 | malX-F  malX-R | GGACATCCTGTTACAGCGCGCA  TCGCCACCAATCACAGCCGAAC | 68 | 922 | BEN2908 | Johnson and Stell [19] |
| *neuC* | Capsule K1 | neu1  neu2 | AGGTGAAAAGCCTGGTAGTGTG  GGTGGTACATTCCGGGATGTC | 61 | 676 | BEN2908 | Moulin-Schouler et al. [16] |
| *ompT* | Episomal outer membrane protease | *ompT-F*  *ompT-R* | TCATCCCGGAAGCCTCCCTCACTACTAT  TAGCGTTTGCTGCACTGGCTTCTGATAC | 63 | 496 | BEN2908 | Johnson et al. [13] |
| *P(F11)* | felA | fel1  fel2 | GGTCAASCAGCTAAAAACGGTAAGG  CCTTCAGAAACAGTACCGCCATTCG | 61 | 239 | BEN2905 | Moulin-Schouler et al. [16] |
|  | papC | pap1  pap2 | GACGGCTGTACTGCAGGGTGTGGCG  ATATCCTTTCTGCAGGGATGCAATA | 61 | 328 | BEN2905 | Le Bouguénec et al. [24] |
| *pic* | Serine protease autotransporter | pic-F  pic-R | ACTGGATCTTAAGGCTCAGG  TGGAATATCAGGGTGCCACT | 60 | 411 | BEN2936 | Ewers et al. [15] |
| *sat* | Secreted autotransporter toxin | sat-F  sat-R | TGCTGGCTCTGGAGGAAC  TTGAACATTCAGAGTACCGGG | 60 | 667 | BEN2936 | Ewers et al. [15] |
| *sfaS* | S adhesin of the type S fimbriae | sfaS-F  sfaS-R | GTGGATACGACGATTACTGTG  CCGCCAGCATTCCCTGTATTC | 63 | 242 | BEN2742 | Johnson and Stell [19] |
| *sitA* | Iron transport protein (=A12) | sitA-F  sitA-R | ATGCACTCGATAAAAAAAGT  TTAAGAAGGTCGATATACGT | 53 | 860 | BEN2908 | Schouler et al. [10] |
| *tia* | Toxigenic invasion locus | tia-F  tia-R | AGCGCTTCCGTCAGGACTT  ACCAGCATCCAGATAGCGAT | 60 | 512 | ECCO 18 | Ewers et al. [15] |
| *traT* | Protectin-transfer and serum resistance protein | tratT-F  traT-R | GGTGTGGTGCGATGAGCACAG  CACGGTTCAGCCATCCCTGAG | 68 | 290 | BEN2908 | Johnson and Stell [19] |
| *tsh* | Thermosensitive haemagglutinin | tsh-F  tsh-R | GGTGGTGCACTGGAGTGG  AGTCCAGCGTGATAGTGG | 55 | 640 | BEN2277 | Dozois et al. [25] |
| TspE4.C2 | Anonymous DNA fragment | TspE4C2.1  TspE4C2.2 | GAGTAATGTCGGGGCATTCA  CGCGCCAACAAAGTATTACG | 59 | 152 | BEN2908 | Clermont et al. [12] |
| *uidA* | *E. coli* beta-glucuronidase | uidA-F  uidA-R | ATGGAATTTCGCCGATTTTGC  ATTGTTTGCCTCCCTGCTGC | 60 | 187 | BEN2908 | Heijnen and Medema [26] |
| *vat* | Vacuolating autotransporter toxin | vat-F  vat-R | GTGTCAGAACGGAATTGTC  GGGTATCTGTATCATGGCAAG | 60 | 230 | BEN2936 | Frömmel et al. [23] |
| *yjaA* | Conserved protein with unkown function | yjaA.1  yjaA.2 | TGAAGTGTCAGGAGACGCTG  ATGGAGAATGCGTTCCTCAAC | 59 | 211 | BEN2908 | Clermont et al. [12] |

Primers used for PCR amplification

**Results**

**Serum bactericidal test**

Figure 1


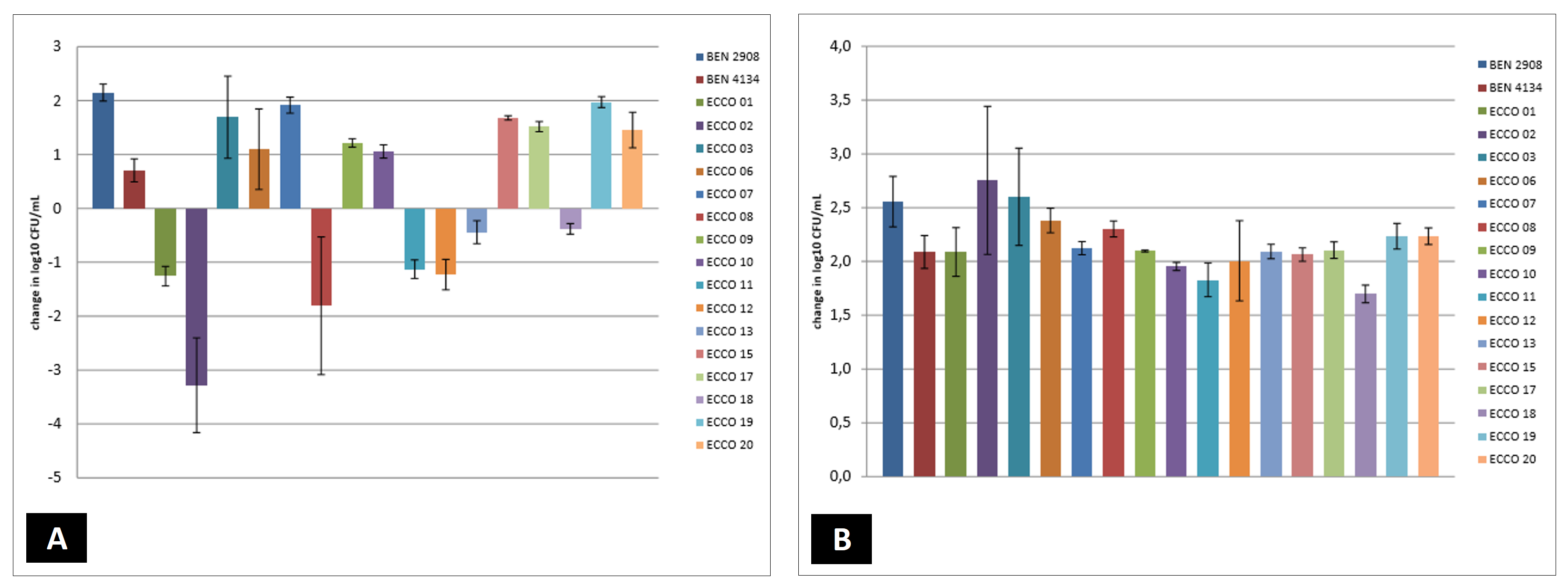


Serum resistance of *E. coli* strains in complet SPF chicken serum (a) and inactivated SPF chicken serum (b).

**References**

1. Clermont O, Johnson JR, Menard M, Denamur E. Determination of *Escherichia coli* O types by allele-specific polymerase chain reaction: application to the O types involved in human septicemia. Diagn Microbiol Infect Dis. 2007;57:129–13.
2. Clermont O, Olier M, Hoede C, Diancourt L, Brisse S, Keroudean M, Glodt J, Picard B, Oswald E, Denamur E. Animal and human pathogenic *Escherichia coli* strains share common genetic backgrounds. Infect Genet Evol. 2011;11:654–662.
3. Fratamico PM, Yan X, Liu Y, DebRoy C, Byrne B, Monaghan A, Fanning S, Bolton D. *Escherichia coli* serogroup O2 and O28ac O-antigen gene cluster sequences and detection of pathogenic *E. coli* O2 and O28ac by PCR. Can J Microbiol. 2010;56:308–316.
4. Li D, Liu B, Chen M, Guo D, Guo X, Liu F, Feng L, Wang L: A multiplex PCR method to detect 14 *Escherichia coli* serogroups associated with urinary tract infections. J Microbiol Methods. 2010;82:71–7.
5. Liu B, Wu F, Li D, Beutin L, Chen M, Cao B, Wang L: Development of a serogroup-specific DNA microarray for identification of *Escherichia coli* strains associated with bovine septicemia and diarrhea. Vet Microbiol. 2010;142:373–8.
6. Bielaszewska M, Mellmann A, Zhang W, Köck R, Fruth A, Bauwens A, Peters G, Karch H: Characterisation of the *Escherichia coli* strain associated with an outbreak of haemolytic uraemic syndrome in Germany, 2011: A microbiological study. Lancet Infect Dis. 2011;11:671–676.
7. Bugarel M, Beutin L, Martin A, Gill A, Fach P: Micro-array for the identification of Shiga toxin-producing *Escherichia coli* (STEC) seropathotypes associated with Hemorrhagic Colitis and Hemolytic Uremic Syndrome in humans. Int J Food Microbiol. 2010;142:318–329.
8. Iguchi A, Iyoda S, Ohnishi M: Molecular characterization reveals three distinct clonal groups among clinical shiga toxin-producing *Escherichia coli* strains of serogroup O103. J Clin Microbiol. 2012;50:2894–900.
9. Dai J, Wang S, Guerlebeck D, Laturnus C, Guenther S, Shi Z, Lu C, Ewers C: Suppression subtractive hybridization identifies an autotransporter adhesin gene of E. coli IMT5155 specifically associated with avian pathogenic *Escherichia coli* (APEC). BMC Microbiol. 2010;10:236.
10. Schouler C, Koffmann F, Amory C, Leroy-Sétrin S, Moulin-Schouleur M: Genomic subtraction for the identification of putative new virulence factors of an avian pathogenic *Escherichia coli* strain of O2 serogroup. Microbiol. 2004;150:2973–84.
11. Yamamoto T, Nakazawz M. Detection and sequences of the enteroaggregative *Escherichia coli* heat-stable enterotoxin 1 gene in enterotoxigenic *E. coli* strains isolated from piglets and calves with diarrhea. J Clin Microbiol. 1997;35:223-7.
12. Clermont O, Bonacorsi S, Bingen E: Rapid and simple determination of the *Escherichia coli* phylogenetic group. Appl Environ Microbiol. 2000;66:4555–8.
13. Johnson TJ, Wannemuehler Y, Doetkott C, Johnson SJ, Rosenberger SC, Nolan LK: Identification of Minimal Predictors of Avian Pathogenic *Escherichia coli* Virulence for Use as a Rapid Diagnostic Tool. J Clin Microbiol. 2008;46:3987–3996.
14. Blanco M, Blanco IE, Blanco J, Alonsob MP, Balsalobre C, Madrid C, Juirez A. Polymerase chain reaction for detection of *Escherichia coli* strains producing cytotoxic necrotizing factor type 1 and type 2 (CNFl and CNF2). J Microbiol Methods. 1996;26:95–101.
15. Ewers C, Li G, Wilking H, Kießling S, Alt K, Antáo EM, Laturnus C, Diehl I, Glodde S, Homeier T, Böhnke U, Steinrück H, Philipp HC, Wieler LH: Avian pathogenic, uropathogenic, and newborn meningitis-causing *Escherichia coli*: How closely related are they? Int J Med Microbiol. 2007;297:163–176.
16. Moulin-Schouleur M, Schouler C, Tailliez P, Kao M-R, Brée A, Germon P, Oswald E, Mainil J, Blanco M, Blanco J: Common virulence factors and genetic relationships between O18:K1:H7 *Escherichia coli* isolates of human and avian origin. J Clin Microbiol. 2006;44:3484–92.
17. Marc D, Dho-Moulin M: Analysis of the fim cluster of an avian O2 strain of *Escherichia coli*: serogroup-specific sites within fimA and nucleotide sequence of fimI. J Med Microbiol. 1996;44:444–52.
18. Arné P, Marc D, Brée A, Schouler C, Dho-Moulin M: Increased tracheal colonization in chickens without impairing pathogenic properties of avian pathogenic *Escherichia coli* MT78 with a fimH deletion. Avian Dis. 2000;44:343–55.
19. Johnson JR, Stell AL: Extended virulence genotypes of *Escherichia coli* strains from patients with urosepsis in relation to phylogeny and host compromise. J Infect Dis. 2000;181:261–72.
20. Schubert S, Rakin a., Karch H, Carniel E, Heesemann J: Prevalence of the “high-pathogenicity island” of Yersinia species among *Escherichia coli* strains that are pathogenic to humans. Infect Immun. 1998;66:480–485.
21. Ewers C, Schüffner C, Weiss R, Baljer G, Wieler L: Molecular characteristics of *Escherichia coli* serogroup O78 strains isolated from diarrheal cases in bovines urge further investigations on their zoonotic potential. Mol Nutr Food Res. 2004;48:504–14.
22. Germon P, Chen YH, He L, Blanco JE, Brée A, Schouler C, Huang SH, Moulin-Schouleur M: ibeA, a virulence factor of avian pathogenic *Escherichia coli*. Microbiol. 2005;151:1179–1186.
23. Fr̈mmel U, Lehmann W, R̈diger S, B̈hm A, Nitschke J, Weinreich J, Groß J, Roggenbuck D, Zinke O, Ansorge H, Vogel S, Klemm P, Wex T, Schr̈der C, Wieler LH, Schierack P: Adhesion of human and animal *Escherichia coli* strains in association with their virulence-associated genes and phylogenetic origins. Appl Environ Microbiol. 2013;79:5814–5829.
24. Le Bouguenec C, Archambaud M, Labigne a.: Rapid and specific detection of the pap, afa, and sfa adhesin-encoding operons in uropathogenic *Escherichia coli* strains by polymerase chain reaction. J Clin Microbiol. 1992;30:1189–1193.
25. Dozois CM, Dho-Moulin M, Brée A, Fairbrother JM, Desautels C, Curtiss R: Relationship between the Tsh autotransporter and pathogenicity of avian *Escherichia coli* and localization and analysis of the tsh genetic region. Infect Immun. 2000;68:4145–4154.
26. Heijnen L, Medema G: Quantitative detection of E. coli, E. coli O157 and other shiga toxin producing E. coli in water samples using a culture method combined with real-time PCR. J Water Health. 2006;04(Suppl 2):487–498.
